# What do we know about health-related knowledge translation in the Circumpolar North? Results from a scoping review

**DOI:** 10.3402/ijch.v75.31223

**Published:** 2016-04-20

**Authors:** M. Ellen McDonald, Andrew Papadopoulos, Victoria L. Edge, James Ford, IHACC Research Team, Alison Sumner, Sherilee L. Harper

**Affiliations:** 1 Department of Population Medicine, University of Guelph, Guelph, ON, Canada; 2 Department of Geography, McGill University, Montréal, QC, Canada

**Keywords:** Inuit, Circumpolar North, knowledge translation, knowledge transfer, knowledge exchange, dissemination, results sharing, health, public health messaging, scoping review

## Abstract

**Background:**

Health research knowledge translation (KT) is important to improve population health outcomes. Considering social, geographical and cultural contexts, KT in Inuit communities often requires different methods than those commonly used in non-Inuit populations.

**Objectives:**

To examine the extent, range and nature of literature about health-related KT in Inuit communities.

**Design:**

A scoping review was conducted. A search string was used to search 2 English aggregator databases, ProQuest and EBSCOhost, on 12 March 2015. Study selection was conducted by 2 independent reviewers using inclusion and exclusion criteria. To be included, studies had to explicitly state that KT approaches were used to share human health research results in Inuit communities in the Circumpolar North. Articles that evaluated or assessed KT approaches were thematically analysed to identify and characterize elements that contributed to KT success or challenges.

**Results:**

From 680 unique records identified in the initial search, 39 met the inclusion criteria and were retained for analysis. Of these 39 articles, 17 evaluated the KT approach used; thematic analysis identified 3 themes within these 17 articles: the value of community stakeholders as active members in the research process; the importance of local context in tailoring KT strategies and messaging; and the challenges with varying and contradictory health messaging in KT. A crosscutting gap in the literature, however, included a lack of critical assessment of community involvement in research. The review also identified a gap in assessments of KT in the literature. Research primarily focused on whether KT methods reflected the local culture and needs of the community. Assessments rarely focused on whether KT had successfully elicited its intended action.

**Conclusions:**

This review synthesized a small but burgeoning area of research. Community engagement was important for successful KT; however, more discussion and discourse on the tensions, challenges and opportunities for improvement are necessary.

The purpose of applied health research is to contribute to the development of actions that will improve the health of populations (1). The creation of new knowledge from research is, on its own, often unlikely to lead to implementation or improvement of population health; therefore, a process to translate knowledge into practical action is necessary (2). Many health research funding agencies have recognized the importance and necessity of the transmission, translation and exchange of new and existing health knowledge between researchers and stakeholders (e.g. other researchers, practitioners, policy-makers and community members) (3–5). Knowledge translation (KT) is a widely used term that describes the attempt to move research results into practical action. There are, however, many other terms used in health research to describe knowledge sharing with similar functions, such as “knowledge transfer,” “knowledge exchange,” “dissemination,” “results sharing,” among others (Table I) (6,7). The concept of KT is broad and commonly involves “end of grant KT,” whereby researchers share the research results with stakeholders at the end of the project (2). More recently, there has been movement towards “integrated KT,” whereby stakeholders are involved throughout the research process (8–10), working together to build the research question, develop the methods and data collection tools, as well as actively engage in data collection, results interpretation and results dissemination (2). For the purpose of this paper, the term KT will serve as an all-encompassing term for those knowledge-sharing activities that involve moving research results to practical action in some capacity, ranging from KT approaches that are integrated throughout the research process (i.e. integrated KT) to research results dissemination (i.e. end of grant KT).

**Table:** *Table I*. Example definitions of knowledge translation–related terms

Term	Definition
Knowledge translation	“a dynamic and iterative process that includes synthesis, dissemination, exchange and ethically-sound application of knowledge to improve the health of Canadians, provide more effective health services and products and strengthen the health care system. This process takes place within a complex system of interactions between researchers and knowledge users which may vary in intensity, complexity and level of engagement depending on the nature of the research and the findings as well as the needs of the particular knowledge user.” – Canadian Institutes of Health Research (CIHR), 2014 (*http://www.cihr-irsc.gc.ca/e/39033.html#Definition*) “the process of translating knowledge from one format to another so that the receiver can understand it; often from specialists to non-specialists. KT is sometimes represented as a one-way, and sometimes a two-way, process” (11, p. 2)**.
Knowledge transfer	“a one-way process of sharing knowledge which can be construed as more of a teacher- student relationship than other knowledge-related activities and perhaps associated with mutual exploration of an issue” (11, p. 2)**.
Knowledge exchange *or* knowledge transfer and exchange	“The exchange of knowledge refers to the interaction between the knowledge user and the researcher, resulting in mutual learning.” – *CIHR, 2014 (* *http://www.cihr-irsc.gc.ca/e/39033.html#Definition* *)*
	“KTE [knowledge transfer and exchange] is a process of exchange between researchers and stakeholders/knowledge users designed to make relevant research information available and accessible to stakeholders for use in practice, planning and policy-making. Stakeholders are involved early in the research process to provide guidance to shape the research questions and information about the context in which research results are likely to be used.” – *Institute for Work & Health, no date (n.d.) (* *http://www.iwh.on.ca/kte-principles* *)*
Dissemination	“Dissemination involves identifying the appropriate audience and tailoring the message and medium to the audience. Dissemination activities can include such things as summaries for/briefings to stakeholders, educational sessions with patients, practitioners and/or policy makers, engaging knowledge users in developing and executing dissemination/implementation plan, tools creation, and media engagement.” – *CIHR, 2014 (* *http://www.cihr-irsc.gc.ca/e/39033.html#Definition* *)* “an active approach of spreading evidence-based interventions to the target audience via determined channels using planned strategies” (12, p. 118).
Results sharing or knowledge sharing	“The exchange of knowledge between and among individuals, and within and among teams, organizational units, and organizations. This exchange may be focused or unfocused, but it usually does not have a clear a priori objective.” “An exchange of knowledge between 2 individuals: one who communicates knowledge and one who assimilates it. In knowledge sharing, the focus is on human capital and the interaction of individuals. Strictly speaking, knowledge can never be shared. Because it exists in a context; the receiver interprets it in the light of his or her own background” – *Encyclopedia of Knowledge Management, 2006* (13) **.
Implementation	“the use of strategies to adopt and integrate evidence-based health interventions and change practice patterns within specific settings.” – *Fogarty International Center, National Institutes of Health*, 2013 (http://www.fic.nih.gov/News/Events/implementation-science/Pages/faqs.aspx) “the process of putting to use or integrating evidence-based interventions within a setting” (12, p. 118).
Diffusion	“the passive, untargeted, unplanned, and uncontrolled spread of new interventions. Diffusion is part of the diffusion-dissemination-implementation continuum, and it is the least-focused and intense approach” (12, p. 118).

While the KT literature is diverse and growing (6,11), there has been comparatively less published about KT in Indigenous contexts (14). The articles that have been published about Indigenous health KT suggest that differential social and cultural mechanisms of communication, along with historical, political and economic contexts within Indigenous populations necessitate unique approaches to KT (9,15–19). Moreover, many argue that the disparities in health outcomes between Indigenous and non-Indigenous populations are, in part, due to challenges in effective KT (12,20–22). Thus, the unique KT approaches required for Indigenous communities, disparities in Indigenous health outcomes and failures in past Indigenous health KT underpin the need for further research in this area (14,21,22).

Within the existing, albeit limited, Indigenous health KT literature, there is a clear deficit in Inuit (i.e. individuals self-identifying as Inupiat, Yupik, Inuit, Inuvialuit, Kalaallit and Yupik (23)) health KT–published literature. Indeed, while health KT literature for Inuit is sparse, there are several publications focusing on KT frameworks, approaches, ethical imperatives and best practices for several other Indigenous groups, including First Nations people in Canada (23–25) and the USA (26), Canadian Metis (27), Indigenous communities in Southern Canada (20), Australian Aborigines (28,29) and Māori in New Zealand (30). Similar to other Indigenous contexts, local uptake of Inuit health research results is particularly important because Inuit continue to experience health inequities compared to non-Inuit (e.g. lower life expectancies, higher infant mortality rates, higher infectious and chronic disease rates, and more mental health and wellness challenges) (31–33). As such, health research KT in Inuit communities is particularly important to move research results into practical action (24,34–39).

It is important to understand how KT approaches in Inuit health research reflect differential cultural, political, economic and social norms to increase local uptake of research results. As such, the purpose of this scoping review was to better understand health research KT conducted within Inuit communities in the Circumpolar North. Specifically, the scoping review characterized the extent, range and nature of existing health-related KT literature in the Circumpolar North.

## Methods

There were 5 key stages in this scoping review: (a) research question identification and search query development; (b) relevant study identification; (b) study selection; (d) data charting; and (e) results collation, summarization, thematic analysis and reporting (40,41).

### Research question development, data sources and search strategy

The research question identified was “What does the literature tell us about knowledge translation in Inuit communities in the Circumpolar North?” To develop a search query to investigate this question, a preliminary search of general KT articles was conducted to identify KT terminology used in published papers and to create a broad list of potentially relevant terms. A library scientist assisted in developing various combinations of terms o develop a search string that included concepts (e.g. KT terms), populations (e.g. Inuit in Circumpolar locations) and outcomes (e.g. health and well-being). The final search string (Table II) was used to search EBSCOHost and ProQuest^®^ aggregator databases with select electronic databases (Appendix 1), including peer-reviewed and grey literature. Searches were conducted on 12 March 2015 and limited to English language publications. The searches were not limited by year (except for restrictions on database capacity). Hand-searching key journals for relevant articles is commonly recommended for scoping reviews (40,41); as such, 3 key journals (*International Journal of Circumpolar Health*, *Arctic* and *Inuit Studies*) were hand-searched for relevant articles published in the past decade (March 2005 to March 2015). All returned citations from database searches were exported into RefWorks^©^ online research management tool and then exported into the systematic review software DistillerSR, where all duplicates were removed.

**Table:** *Table II*. Search string used in EBSCOHost and ProQuest^®^ aggregator databases including terms for population, knowledge translation, health and location

Category	Terms
Population	Inuit OR Inuk OR Inupiat OR Inupiat OR Inuvialuit OR Eskimos OR Tikigaq OR Inuvialuit OR Netsilik OR Eskimo OR Kalaallits OR Kalaallit OR Aleuts OR Aleut OR Yupik OR Yup'ik OR Alutiiq OR Chugach
KT	“knowledge translation” OR “knowledge transfer” OR “knowledge mobilization” OR “results sharing” OR “results dissemination”
Health	Health OR well-being OR wellness OR aid OR disorder OR burden OR preventative OR chronic
Location	Circumpolar OR Arctic OR Canada OR Denmark OR Greenland OR Chukotka OR Russia OR Alaska

### Eligibility criteria

Articles that reported using KT approaches to share human health research results in Inuit communities in the Circumpolar North were included in this scoping review. The World Health Organization's definition of health was used to define human health, which involves a holistic understanding of health (i.e. physical, mental and social well-being) and not simply the absence of disease (42). Therefore, the health research topic had to include an investigation of the physical, mental or social well-being of humans. Research results being shared about the environmental impact on human health were considered but those focused solely on the health of the environment were excluded. Studies had to focus on peoples originating from Thule culture, using the Inuit Circumpolar Council definition, including Inupiat, Yupik (Alaska), Inuit, Inuvialuit (Canada), Kalaallit (Greenland) and Yupik (Russia) (23). Furthermore, the articles had to engage Inuit in the KT activity itself (i.e. Inuit were transferring, exchanging or receiving knowledge). Strategies for KT with solely non-Inuit target audience (e.g. academic poster presentations targeting southern scientists) and studies that used pan-Indigenous (e.g. combining First Nation, Métis and Inuit populations together) instead of Inuit-specific approaches were excluded. Geographically, studies conducted in countries in the Circumpolar North were considered, which included parts of Canada, Alaska (United States), Greenland and Chukotka (Russia) (23).

### Relevance screening

A two-stage screening process was conducted by 2 independent reviewers to identify relevant articles. First, the title and abstracts of all returned citations were screened based on the eligibility criteria using a form developed prior to the review. The relevant citations proceeded to the second stage, where the full text of the article was reviewed based on the eligibility criteria using a form developed prior to the review. Conflicts were discussed between reviewers; if a consensus could not be reached, a third reviewer decided if the study would be included. The degree of agreement between reviewers was assessed (i.e. inter-rater agreement via Cohen's Kappa (κ)) for both stages of screening (i.e. title/abstract and full text). The hand-search of the 3 key journals used the same inclusion/exclusion criteria as database searches. When the same study was published twice (e.g. a thesis dissertation and journal article of the same study), the article with the most information about KT was retained for analysis, and the duplicate article was discarded.

### Data extraction & synthesis

Data were extracted from the included studies using a charting form to capture both count data, as well as descriptions of study results (41). Information extracted included author(s), year of publication, year of study, location of study within the Circumpolar North, types of documents (journal article, thesis), specific health subfield and health outcome studied (Table III). The methodology for KT was also charted, including the KT method used, method of KT assessment and outcome measures of KT success. To identify and characterize elements that contributed to KT success and/or challenges, qualitative analysis was conducted for those studies that *evaluated* the KT approaches used to share human health research results in Inuit communities in the Circumpolar North. Specifically, to be included in the qualitative analysis, the KT activities had to be assessed (e.g. quantitative and/or qualitative evaluations, and/or reflections) in the study. For instance, several studies described the KT process but did not evaluate the process, and these studies were excluded from the qualitative analysis. Thematic analysis was conducted, which involved familiarization with the article content; generating initial codes; and developing, refining and defining themes (43).

**Table:** *Table III*. Summary of information collected in data charting form during the data extraction step of this scoping review included knowledge translation (KT) methodology, article information and overall recommendations given by the studies

Charting categories	Information collected
Information on the article	• Author(s) • Year of publication • Year of study • Location within Circumpolar North • Type of document • Target group context of article written • Specific health subfield • Health outcome studied • Types of researchers/organizations involved
KT methodology	• Aim(s) of study • Study design • KT method used • Validation of KT • Method of KT assessment • Outcome measures of KT success
Recommendations	• Important results

## Results

### General characteristics of the articles

**Fig. 1.**
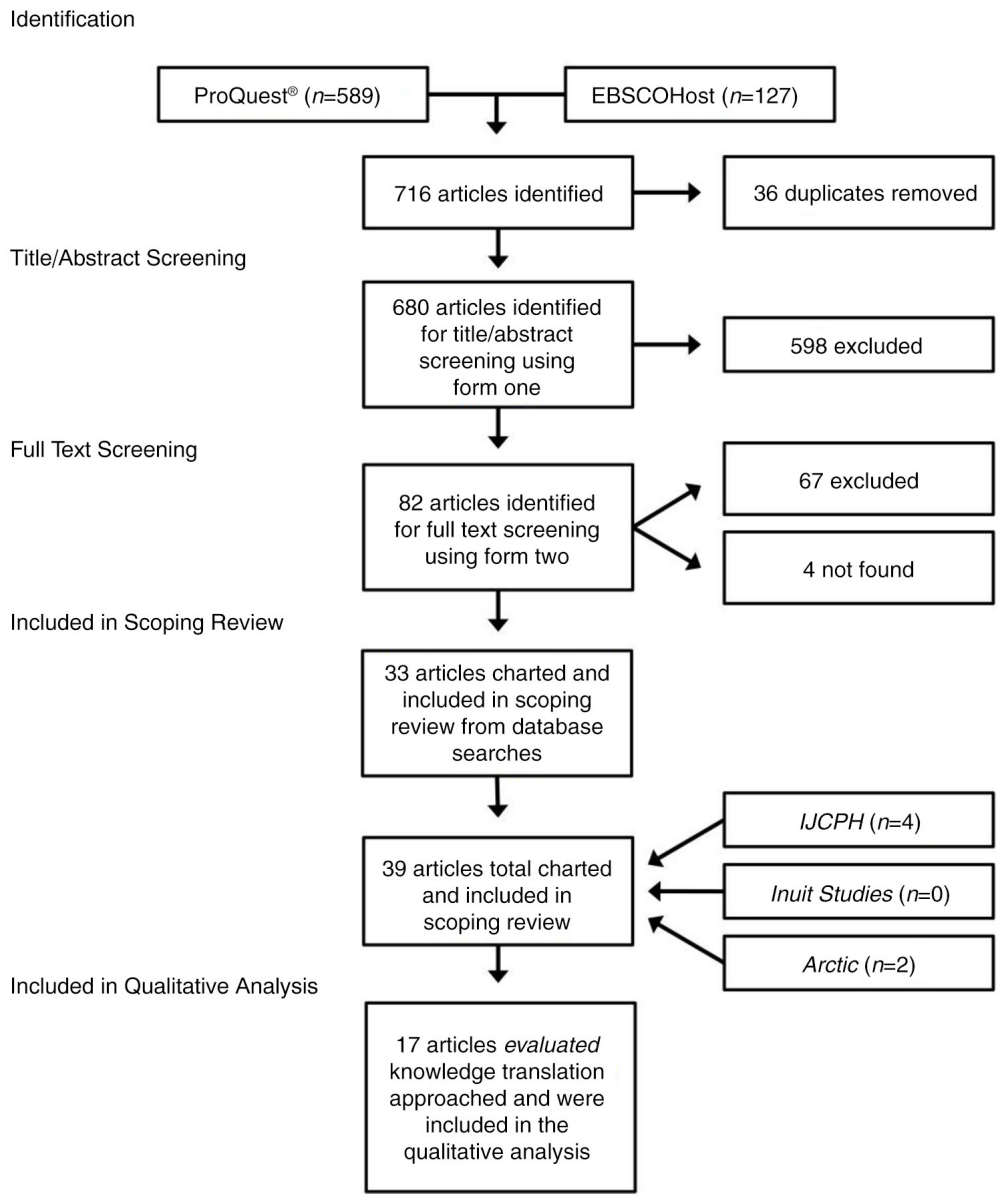
Search result numbers in the form of a flow diagram including identification, title/abstract screening, full article screening, as well as included articles in the scoping review according to the Preferred Reporting Items for Systematic Reviews and Meta-Analyses (PRISMA) flow diagram for reporting standards in systematic reviews (44).

From 680 unique records identified in the initial search, 39 articles met the inclusion criteria and were retained for quantitative analysis (Fig. 1). There was strong agreement between reviewers at the title/abstract level (inter-rater reliability was high at κ = 0.82) and full-article screening level (inter-rater reliability was high at κ = 0.84). Herein, we outline the general characteristics of the 39 included articles.

### Most studies were published in the past decade and were primarily from Canada

**Fig. 2.**
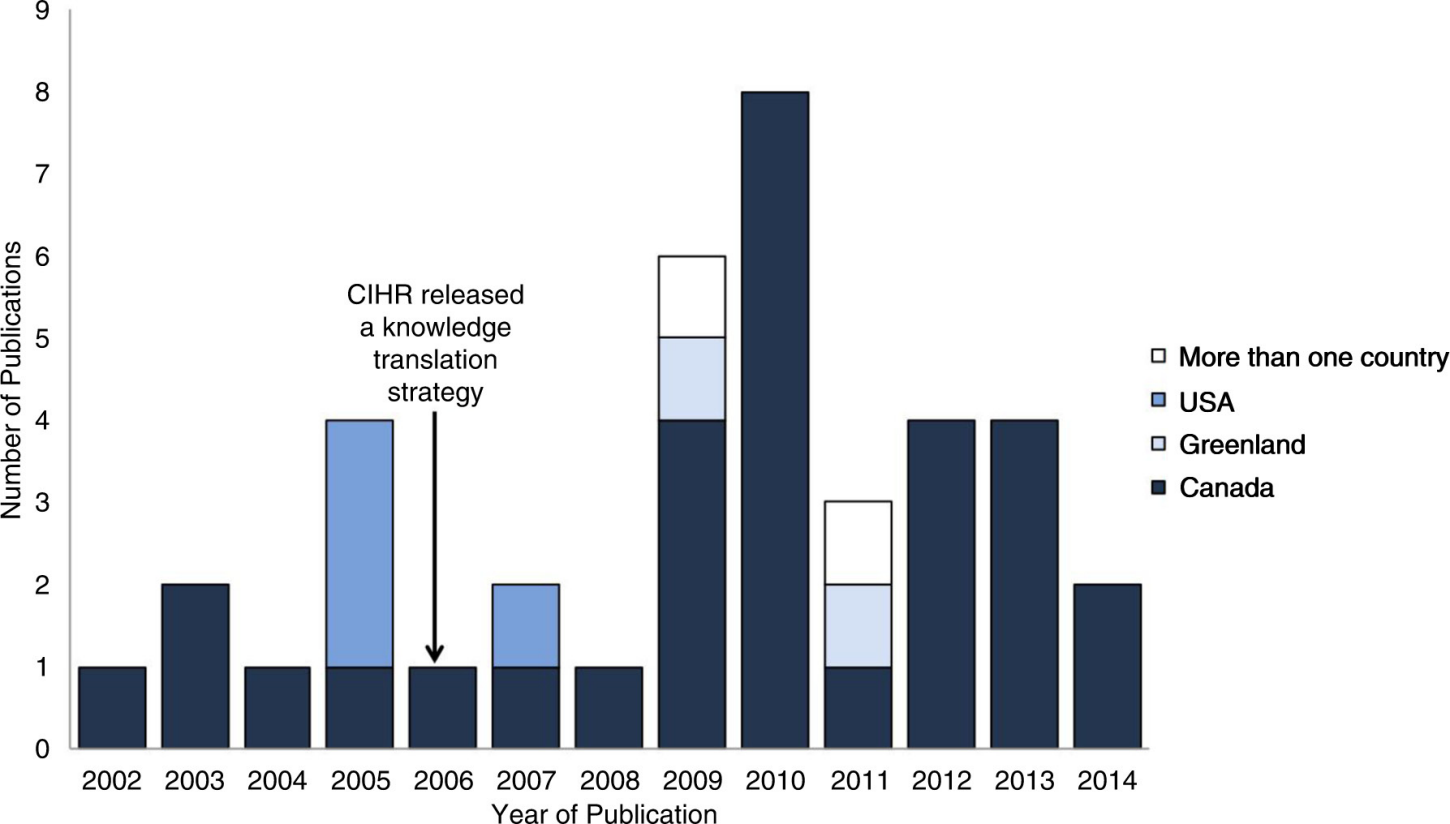
Location and year of the study, with an arrow indicating the year that Canadian Institutes of Health Research (CIHR) released its Knowledge Translation Strategy (82).

Thirty-one (80%) of the articles included were conducted in Northern Canada (45–74), 4 (10%) in Alaska, United States (75–78), 2 (5%) in Greenland (31,79) and 2 (5%) were international in focus (33,80). All studies were published between 2002 and 2014 (Fig. 2). A wide variety of health outcomes were discussed in the studies, including, but not limited to, environmental health (38,40,81), diet and nutrition (24,30,60), drinking water (31,43), zoonotic parasites (48), substance misuse (i.e. drugs and alcohol) and foetal alcohol spectrum disorder (FASD) (51), unintentional injury (40,54,63), lifestyle behaviours (34,35,60), contaminants (52–54), sexual health (31) and heart and vascular disease (75), as well as genetics (76,77).

Of the 39 included articles, 17 articles (44%) performed some form of evaluation or assessment of the KT approaches mentioned in this paper (Table IV). These 17 articles were further examined in order to identify and characterize the elements that contributed to KT success and/or challenges. Herein, we describe the general characteristics of the evaluations conducted in these 17 articles, as well as the findings from the qualitative thematic analysis.

**Table:** *Table IV*. Select findings from charting of articles that *evaluated* knowledge translation (KT) including author(s), title, document type, year study was published, year study was conducted, field of health research, location, type of KT assessment and funding source(s)

Authors	Title	Document type	Publication year	Study year	Field of health research	Location	Type of KT assessment	Funding source(s)	Ref.
Dean, L.S.	Environment and health risk communication pathways in Aboriginal communities: Learning from the case of foodweb contaminants and nutrition issues with young Inuit women in Nunatsiavut	Thesis	2009	2005	Environmental health risk messages	Nain, Nunatsiavut, Newfoundland & Labrador, Canada	Qualitative evaluation	Canadian Institutes of Health Research (CIHR), Northern Contaminants Program (Indian and Northern Affairs)	(45)
Yohannes, S.	Traditional food consumption, anthropometry, nutrient intake and the emerging relationship between Inuit youth and traditional knowledge in a Baffin Island community.	Thesis	2009	2008	Food use, nutrient status, and anthropometry	Pangnirtung, Nunavut, Canada	Quantitative and qualitative evaluation	Nasivvik Centre (CIHR)	(46)
Kot, M.	Challenges and opportunities for small community drinking water systems – A knowledge translation perspective	Thesis	2009	N/A	Drinking water	Anonymous community, Nunavut, Canada	Qualitative evaluation	Natural Sciences and Engineering Research Council (NSERC)	(47)
Pufall, E.L. et al.	Community-derived research dissemination strategies in an Inuit community	Journal Article	2010	2009	Zoonotic parasites in wildlife meat	Nain, Nunatsiavut, Newfoundland & Labrador, Canada	Qualitative evaluation	Nasivvik Centre (CIHR), International Polar Year	(48)
Hamel, C.	Determinants of Participation in an Online Community of Practice (OCoP)	Thesis	2010	2008	Epidemiology & community-based research initiatives	Nunatsiavut in Newfoundland and Labrador, Nunavik in Quebec, Nunavut, the North West Territories, and Ottawa, Canada	Qualitative evaluation	N/A	(49)
Counil, E. et al.	Translational research to reduce trans-fat intakes in Northern Quebec (Nunavik) Inuit communities: a success story?	Journal article	2012	2007–2009	Dietary trans-fat	Kuujjuaq and Akulivik Nunavik, Canada	Reflection only	Nasivvik Centre (CIHR), International Polar Year	(50)
Poole, N. et al.	Improving substance use treatment for First Nations, Métis and Inuit Women: Recommendations arising from a virtual inquiry project	Journal article	2013	2010–2011	Substance use and foetal alcohol spectrum disorder (FASD)	Nunavut, and Northern Quebec, Canada	Reflection only	Health Canada	(51)
Jardine, C. et al.	Knowledge translation with Northern Aboriginal communities: A case study	Journal article	2010	N/A	Health risks: lifestyle behaviours, exposure to environmental contaminants	Nain and Hopedale, Nunatsiavut, Newfoundland & Labrador, Canada	Reflection only	Health Canada	(52)
Tyrrell, M.	Making sense of contaminants: A case study of Arviat, Nunavut	Journal article	2006	N/A	Contaminants	Arviat, Nunavut, Canada	Reflection only	N/A	(53)
									
Myers, H. et al.	Long-Range Transport of Information: Are Arctic Residents Getting the Message about Contaminants?	Journal article	2005	2002–2003	Contaminant knowledge	Clyde River, & Pond Inlet, Nunavut; Makkovik, & Nain, Labrador, Canada	Quantitative and qualitative evaluation	Northern Contaminants Program (Indian and Northern Affairs)	(54)
Gesink, D. et al.	Developing a culturally competent and socially relevant sexual health survey with an urban Arctic community	Journal article	2009	N/A	Sexual health	Nuuk, Greenland	Quantitative and qualitative evaluation	Greenlandic Medical Research Council, CIHR Team in Circumpolar Chronic Disease Prevention, Kommisionen for Videnskabelige Undersøgelsen I Grønland	(31)
Ebbesson, S.O.E. et al.	Recruitment and community interactions in the GOCADAN Study	Journal article	2005	2000–2004	Heart and vascular disease	Norton Sound, Alaska, United States	Reflection only	The US National Institutes of Health (NIH)	(75)
Boyer, B.B. et al.	Building a community-based participatory research center to investigate obesity and diabetes in Alaska Natives	Journal article	2005	N/A	Genetics, nutritional and behaviour risk	Southwest Alaska, United States	Reflection only	NIH	(76)
Boyer, B.B. et al.	Sharing results from complex disease genetics studies: A community based participatory research approach	Journal article	2007	N/A	Genetics	Southwest Alaska, United States	Reflection only	NIH	(77)
Thomas, D.	Development of Coastal climate change action plan, Arviat Nunavut	Thesis	2008	2003	Environmental health	Arviat, Nunavut, Canada	Reflection only	Fisheries and Oceans Canada, Manitoba Hydro, Ocean Management Research Network, National Resource Institute	(55)
Bradshaw, C.	Sailivik: A place of tranquility	Thesis	2009	2008	Landscape architecture & mental health	Pangnirtung, Nunavut, Canada	Reflection only	N/A	(56)
Durkalec, A.	Understanding the role of environment for Indigenous health: A case study of sea ice as a place of health and risk in the Inuit community of Nain, Nunatsiavut	Thesis	2013	2010	Environmental health	Nain, Nunatsiavut, Newfoundland & Labrador, Canada	Reflection only	ArcticNet, Nasivvik Centre for Inuit Health and Changing Environments (CIHR)	(57)

### Methods used to assess KT were often informal

**Fig. 3.**
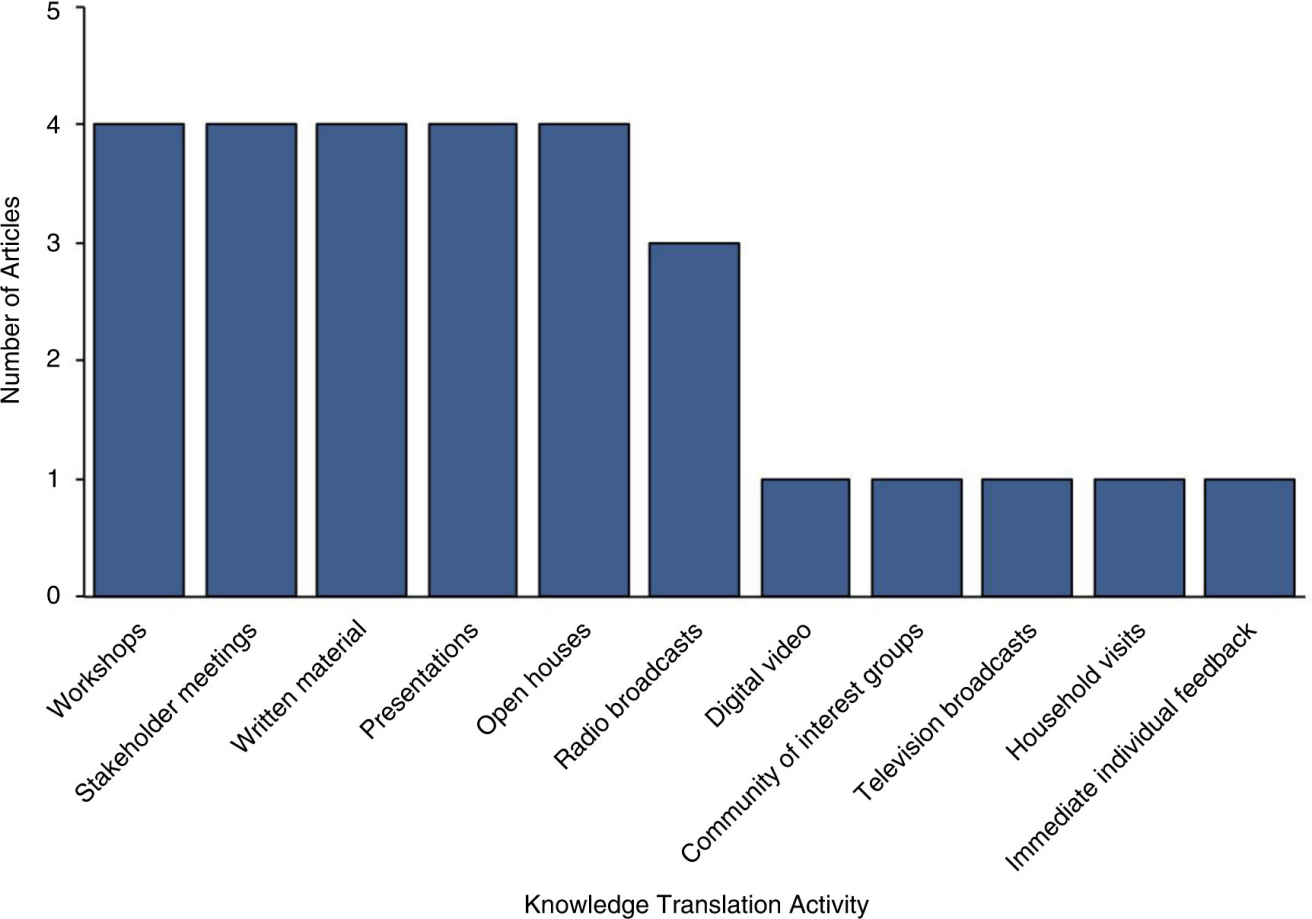
Bar graph indicating the knowledge translation (KT) methods that were assessed in the literature including workshops (53,54,73,77), stakeholder meetings (50,51,53,75), written material (50,53,54,75), presentations (50,57,75,76), open houses (52,54,55,57), radio broadcasts (50,53,57), digital video (46), community of interest groups (47), television broadcasts (53), household visits (75) and immediate individual feedback (categories are not mutually exclusive) (76).

There were a wide variety of KT techniques outlined. Fourteen studies (82.4%) used oral KT methods (e.g. radio, presentation, open house and meeting) in conjunction with, or instead of, written KT methods (e.g. poster and report) (Table IV, Fig. 3) (45,46,48,50–57,75–77).

**Table:** *Appendix 1*. Databases included in EBSCOHost and ProQuest^®^ searches on March 12, 2015.

Aggregator database	Databases included	Results	Databases not included
ProQuest	Alt-PressWatch, Algology Mycology and Protozoology Abstracts (Microbiology C), Animal Behavior Abstracts, Aquatic Science & Fisheries Abstracts (ASFA) 1: Biological Sciences & Living Resources, Aquatic Science & Fisheries Abstracts (ASFA) Aquaculture Abstracts, Aquatic Science & Fisheries Abstracts (ASFA) Marine Biotechnology Abstracts, Bacteriology Abstracts (Microbiology B), Biochemistry Abstracts 1, Biochemistry Abstracts 3, Biotechnology Research Abstracts, Calcium & Calcified Tissue Abstracts, Chemoreception Abstracts, COS Conference Papers Index, Ecology Abstracts, Endocrinology Abstracts, Entomology Abstracts, Genetics Abstracts, Health & Safety Science Abstracts, Human Genome Abstracts, Immunology Abstracts, Industrial and Applied Microbiology Abstracts (Microbiology A), Neurosciences Abstracts, Nucleic Acids Abstracts, Oncogenes and Growth Factors Abstracts, ProQuest Deep Indexing: Biological Sciences, Toxicology Abstracts, TOXLINE, Virology and AIDS Abstracts, Canadian Newsstand Complete, Canadian Research Index, Dissertations & Theses @ University of Guelph, EconLit, Environmental Sciences and Pollution Management, Ethnic NewsWatch, GenderWatch, International Bibliography of the Social Sciences (IBSS), Linguistics and Language Behavior Abstracts (LLBA), PAIS International, Periodicals Archive Online, PILOTS: Published International Literature On Traumatic Stress, ProQuest Sociology Collection, Social Services Abstracts, Worldwide Political Science Abstracts	0	ABI/INFORM Global, ABI/INFORM Trade & Industry, AGRICOLA, ARTbibliographies Modern (ABM), Avery Index to Architectural Periodicals, Plant Science, CBCA Complete, ebrary^®^ e-books, ebrary^®^ e-books, ERIC, Hoover's Company Profiles, International Bibliography of Art (IBA), MLA International Bibliography, Philosopher's Index, PRISMA (Publicaciones y Revistas Sociales y Humanísticas), ProQuest Agriculture Journals, ProQuest Asian Business & Reference, ProQuest European Business, ProQuest Historical Newspapers: The Globe and Mail, ProQuest Historical Newspapers: The New York Times, ProQuest Historical Newspapers: The Scotsman, RILM Abstracts of Music Literature
	MEDLINE^^®^^, Canadian Newsstand Complete, Dissertations & Theses @ University of Guelph, Ethnic NewsWatch, GenderWatch, ProQuest Dissertations & Theses A&I, ProQuest Political Science, ProQuest Psychology Journals	124	
EBSCOHost	America: History & Life, Anthropology Plus, Communication Abstracts, Ergonomics Abstracts, Family & Society Studies Worldwide, GreenFILE, Library Literature & Information Science Full Text (H.W. Wilson), Library, Information Science & Technology Abstracts, Film & Television Literature Index with Full Text	0	Agricola, Art Abstracts (H.W. Wilson), Art Index Retrospective (H.W. Wilson), Business Source Complete, Criminal Justice Abstracts with Full Text, eBook Collection (EBSCOhost), Garden, Landscape & Horticulture Index, Historical Abstracts, Hospitality & Tourism Index, L'Année philologique, Mental Measurements Yearbook with Tests in Print, Music Index, Regional Business News, SPORTDiscus with Full Text, Index Islamicus, eBook Academic Collection (EBSCOhost), FRANCIS, Hospitality & Tourism Complete
	Academic Search Premier, Bibliography of Native North Americans, CINAHL Plus with Full Text, Communication & Mass Media Complete, Environment Index, Women's Studies International, CINAHL	589	

**Fig. 4.**
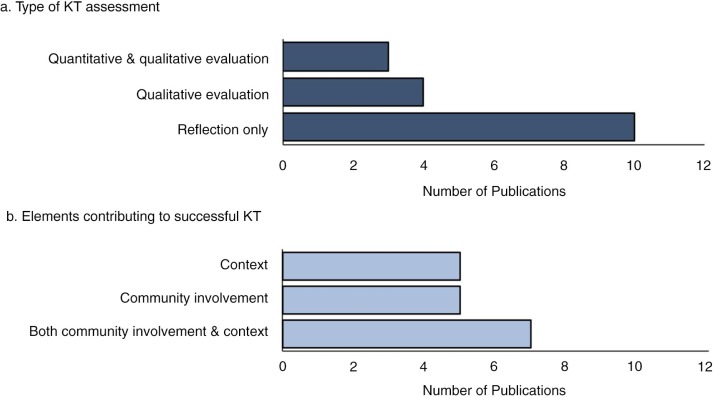
Graphs displaying (a) the type of knowledge translation (KT) assessment (reflection (50–53,55–57,75–77), qualitative evaluation alone (45,47–49) and both quantitative and qualitative methods (46,54,76)), and (b) the elements that the author attributed to KT success (necessity of community involvement (46,50,51,56,76), context (45,47,49,53,54) or both (31,48,51,52,57,75,77)).

Of the articles that performed some form of evaluation or assessment of KT, the assessment activities ranged from reflections on the KT process (e.g. author of the article commented on what did or did not work in the KT strategy used in their study without any formal assessment), to quantitative evaluations (e.g. Likert scale questions, independent and paired *t*-tests) (Fig. 4a). Reflection on the KT strategy was used as the sole assessment tool in 10 studies (58.8%) (50–53,55–57,75–77). Qualitative evaluation were used in 4 (23.5%) studies (45,47–49), and mixed quantitative and qualitative evaluations were used in 3 (17.6%) (46,54,76) studies.

### Most studies assessed the social, political and cultural relevance of the KT method

Thirteen (76.5%) studies assessed whether KT methods fit with social, political and cultural needs or preferences of communities (31,45–49,51,52,55–57,76,77). For example, one study found that stakeholder interviewees preferred KT methods that were interactive, oral–visual and presented in both English and Inuktitut (48). Seven (41.2%) studies assessed whether their health research results had effectively moved to action through their assessment of the extent of local uptake and implementation of policy outcomes (31,45,46,54,57). For example, local uptake of information was assessed in one study, which formally evaluated whether Elder stories on DVD were successful in transferring traditional knowledge about nutrition to youth using a comparison of pre- and post-DVD questionnaires distributed to youth (e.g. independent and paired *t*-test, content analysis and Likert scale interpretation) (46).

### Thematic analysis results

The thematic analysis of the 17 articles that evaluated KT methods resulted in 3 themes (detailed below): (a) community stakeholders as active members in the research process improved KT; (b) context was important in tailoring KT strategies and messaging; (c) varying and contradictory messaging reduces KT success.

#### Theme 1: Community stakeholders as active members in the research improved KT

All studies indicated the necessity of community involvement (29.4%) (46,50,55,56,76), importance of context (29.4%) (45,47,49,53,54), or both (41.2%) (31,48,51,52,57,75,77) for successful KT in Inuit communities in the Circumpolar North (Fig. 4b). Involvement of community stakeholders in the original research was described as a contributing factor to the success of integrated KT throughout the research process in 12 (70.6%) studies (31,46,48,50–52,56,57,75–77). One study informally reflected that the development of Inuit ownership of the research project seemed to increase KT success (56). Another study reflected that the overall success of integrated KT was attributed to developing trusting partnerships with stakeholders, which appeared to allow researchers to anticipate and avoid potential problems and misunderstandings (76). Indeed, other studies echoed similar findings, reflecting that their partnership with community-based researchers was central to the success of KT efforts, increasing the validity and relevance of research results and improving the potential for local uptake of research (48,52,55,57). For instance, one study reflected that consultation with community stakeholders helped improve appropriateness of both the content and delivery of KT (52). Studies reported that approaches to KT were strengthened when local stakeholders directed the KT process (45,46,48). For example, one study indicated that the involvement of youth in the communication of research results was perceived to increase local information uptake (48). While many studies discussed the importance of community involvement in KT (31,46,48,50–52,55–57,75–77), they did not evaluate the impact, if any, that this approach had on local uptake of research results. Furthermore, minimal analysis of the tensions and challenges (if any), or opportunities for improvement were included in the KT assessments. None discussed a decision-making process to decide when, and how much community involvement should occur, or to decide which community stakeholders should be involved. None assessed the appropriateness of varying involvement of community stakeholders and the effect on successful KT. Yet, there were obvious variances in the type and level of community involvement between studies based on the nature of the project and purpose of the KT.

#### Theme 2: Context was important in tailoring KT strategies and messaging

Attention to local cultural, social and geographical context, and target audiences were identified as an important consideration for successful KT in 12 studies (70.6%) (31,45,47–49,51–54,57,75,77). For example, young females were interviewed about past and future food contaminant messaging, and identified that “if the material was deemed irrelevant to their setting and the food choice options they have, it may be disregarded no matter how clear or otherwise balanced the message is” (29, p. 105). The study explained that KT efforts may be ineffective if local challenges are not understood (e.g. public health messaging that instructs young women to not eat a particular food when other options are limited; or, instructing women to not eat a particular food that has important cultural implications) (45). The young female interviewees also suggested that attention to social context (influential KT brokers, sharing networks, pathways of communication used in a particular subgroup) plays a role in successful KT strategies; in particular, many informal pathways (family, friends) were preferred for transmission of nutritional issues (45). Indeed, another study found that youth were influential KT brokers as focus groups and key informant interviewees preferred dissemination strategies where “children were identified as key elements of KT efforts because their involvement in the delivery of research results would increase impact of the message due to their influential role in the community, especially their parents” (31, p. 107–108).

Geographical location was described in one study as an aspect of local context that must be considered in some KT processes (47). Community stakeholder interviewees identified that the remoteness of target communities was a barrier to effective KT, especially for KT-involving training programmes (47). Interviewees identified distance, weather and lack of roads in the community as major obstacles in holding training courses and suggested adaptations to in-person KT approaches, such as training videos or call centres, which may help to overcome these barriers (47).

#### Theme 3: Varying and contradictory messaging reduces KT success

A challenge discussed in 3 (17.6%) of the studies was the lack of consolidated of KT efforts between research groups and organizations, leading to varying and sometimes contradictory messaging causing confusion among target audiences (45,50,54). For instance, one study conducted a survey to evaluate the degree to which community stakeholders had been exposed to and comprehended messages about contaminants in country food (54). The study found that the information had not been broadly received due, in part, to changing and conflicting messages from KT efforts over a period of several years (e.g. “contaminants are bad for them; but country food is the best food; they are being ‘poisoned’ by southern pollution, but are blessed by a strong, nutritious, culturally valued, country food tradition” (36, p. 57)). Furthermore, another study concluded that outreach messaging was more successful, clearer and easier to understand when organizations presented joint messages that reinforced one another, instead of contradicting one another (45).

## Discussion

Internationally, there is an increasing number of peer-reviewed publications that evaluate health-related KT (7). However, we identified a small number of Inuit health publications reporting KT approaches and even fewer Inuit health KT evaluations. In health research, conducting and publishing KT evaluations are critical to learn from past successes or failures in KT. Researcher reflections on their own experience with KT methods can be useful for developing KT strategies; however, critical assessment of tensions and challenges (if any), or opportunities for improvement in various stages throughout KT is necessary. The results of this scoping review speak for the need of more studies that evaluate the efficacy of KT in the Circumpolar North.

The literature identified in this scoping review primarily focused on the importance of considering the local context in KT approaches; however, few evaluations of KT in this review measured or critically evaluated local uptake of research, or the efficacy of moving research results to practical action in some way. As such, future evaluations should focus on formally evaluating whether KT successfully elicited its intended action. For instance, evaluations could investigate whether KT efforts impacted policy, health behaviour, medical practice or public health practice. There is still much to learn about moving research results into practical action, especially in Inuit communities where little is known about local uptake of research results within Inuit knowledge sharing systems and ways of knowing (14,21,52).

The timing and large proportion of studies conducted in Canada could be due partially to endorsement of KT from major Canadian government agencies such as Canadian Institutes of Health Research (CIHR) (31,45–48,50,57,83) and Health Canada (51,52). Indeed, the increase of published KT literature in Canada began 1 year after the CIHR released a Knowledge Translation Strategy in 2004 with specific objectives to fund grants that support KT research (82). Furthermore, another study stated that “CIHR is recognized within Canada and internationally for leading and funding the advancement of KT science and practice” (54, p. 1), which may, in part, explain the predominance of Canadian papers identified in this review. While fewer studies were captured from the United States (75–77) and Greenland (31), KT is still recognized as important by the U.S. National Institutes of Health (75–77) and Greenlandic Medical Research Council (31). Few studies from Russia, USA and Greenland do not necessarily indicate a dearth of KT assessments in these regions; this result may have been due to our English-only search strategy, and/or a lack of peer-reviewed publications of such assessments from these countries.

Our results indicated that KT is more effective when community stakeholders are active members in the research process. Indeed, research in Indigenous communities that is conducted by non-Indigenous researchers can sometimes be met with scepticism and resentment by community members (24,84–88). This potential resentment can be understood by reflecting on decades of research on Indigenous peoples, where some non-Indigenous researchers historically “parachuted” into communities to collect data (often without consent) with limited attempts to involve community stakeholders before, during or following the research process, in the form of KT (24,84–88). Attempts have been made to improve research methods by focusing on community participation, capacity development and social equity (84,87); however, there continue to be embedded power dynamics such as conflicting goals between universities and communities, hierarchy of scientific knowledge over Indigenous knowledge and the production of knowledge by researchers that has limited direct value to the community (24,84–88). In comparison to written or less interactive KT, we found that the interactive oral KT was most commonly used in Inuit communities and was supported by the extended literature as a preferred means of engagement by Inuit and other Indigenous groups (89,90). Future health research projects in Inuit communities should consider the incorporation of oral methods when building KT strategies as Inuit have a strong oral history and culture. While oral methods are appropriate for cultural and historical reasons, they may not be sufficient in moving research results into practical action due, in part, to embedded power dynamics between researchers and research participants (91,92).

There are a number of different approaches available to reduce power differentials in research (93,94). For instance, narrative or story-based approaches to data collection can reduce power imbalances and could be expanded to approaches in KT (87), providing a method for giving voice to Indigenous communities through sharing stories (85,87,95). However, influence from non-Inuit researchers on the stories shared through narrative or story-based approaches (e.g. selection and interpretation of stories shared) can perpetuate these power dynamics despite attempts to avoid this; as such, they should be used with caution (87). One way researchers have attempted to diminish power dynamics in data collection is through methods such as digital storytelling, where Inuit are able to create and control their own research framework, in their own voices (87). If methods such as digital storytelling can decrease power imbalances in primary data collection, then perhaps such methods could also reduce power imbalances in KT, whereby knowledge is generated by the community and disseminated through methods primarily locally led. It may be necessary to rebuild the KT methodological framework for Inuit communities, such that it is grounded in Inuit oral histories and cultural practices, and based on actively attempting to deconstruct power dynamics in the KT process between researchers and research participants and between Inuit and non-Inuit (87).

Community participation as a driver for successful KT was a theme echoed by the findings of van der Velde et al., in their assessment of participatory action research (PAR) in the context of multiethnic mental health research and KT (96). The researchers examined the degree of success relative to the 4 main tenets of PAR – empowerment, social change, participation and learning (or KT) – and found that success in each of the tenets was dependent on the level of community stakeholder participation in the research process (96). Participation was viewed as the “gateway” for engaging in PAR, while successful KT, opportunities for learning and empowerment supported continued engagement in the research process (96). The inclusion of community stakeholders in the study process allowed integrated, and often informal, KT between external and internal community researchers, which can aid in the integration of community values, knowledge and technical assessments in KT processes (97). This engagement with community researchers can lead to a more informed and culturally relevant KT strategy, which promotes successful dissemination and utilization of research (90). In a study that examined factors that influence environmental health KT processes in Canadian Indigenous communities (i.e. Inuit, First Nations and Metis), decision-making actors identified participatory processes as fundamental to successful dissemination and utilization of research (90). Recently, literature has questioned whether or not engagement of community stakeholders in the entire research project is appropriate in *all* projects at *all* times (98–100). There is a need to move beyond uncritically advocating for community engagement and move towards discussion and discourse on the tensions, challenges and opportunities for improvement in community engagement (100). Similarly, assessments of KT should include a critical discussion of the role of community engagement and its effect on whether the KT outcome was achieved. Guidelines regarding when, how and which community members should be involved in KT research should be developed to create strategies for appropriate community engagement.

There are strong arguments for Indigenous health research that is self-determined (owned, controlled, accessed, possessed) by Indigenous communities (101); however, this review only captured one study that explicitly indicated that the community had taken ownership over the entire research process, including KT. One method of facilitating community ownership of KT processes throughout the research project is through the use of negotiated Indigenous research agreements as a natural extension of participatory research (9,102). For instance, the Canadian Tri-Council (102), as well as the Australian Institute of Aboriginal and Torres Strait Islander Studies (9), created a set of guidelines for conducting research with Indigenous partners, whereby complete ownership of the data by Indigenous partners is considered a major principle. These agreements can be advantageous to KT in some communities; however, for other communities these types of formal research negotiations may hold negative connotations stemming from periods during colonization when unethical signing of contracts took place (103). In communities where formal signed research negotiations hold negative connotations, oral negotiations may be more appropriate (103). It is important to ensure clarity of KT expectations (before, during and after the study) of all concerned (funders, researchers, community). It is essential that research bodies in the Circumpolar North continue to commit to equitable research including KT with Indigenous communities, by thinking critically about equitable ownership of research production and dissemination.

Our study found that a comprehensive understanding of the context of target subgroup(s) increased the likelihood of finding appropriate KT methods. The aim of both health promotion and KT is to use health knowledge to elicit an action from community stakeholders. Examination of the literature on health promotion may help to enhance KT strategies to improve moving research results into practical action. The need to tailor KT strategies for individual Inuit communities and subgroups within Inuit communities can be understood through health behaviour models. Many health behaviour models explain that local health promotion can be greatly affected by community and personal context, and that health behaviours are determined by personal perceptions about a disease and the strategies available to decrease its occurrence (104). We can apply many concepts from health behaviour and public health promotion to KT. For instance, subgroup and individual uptake of research results through KT may be explained by the perceived seriousness of disease, susceptibility to disease, and benefits and barriers to behavioural change (104). Perceptions about a disease and the strategies available to decrease its occurrence can be very different between subgroups and can be modified by cues to action (e.g. events, people or things that motivate behavioural change), motivating factors (e.g. culture, education level and past experiences) and self-efficacy (i.e. belief in one's own ability to do something) (104). Strategies for KT may be more effective if they are grounded in a health behaviour model and incorporate understandings of local or target subgroup cues to action, motivating factors and self-efficacy. If KT messages are able to capture the particular Inuit community or subgroup's context, and elicit social modelling, KT may be more successful in influencing health behaviour within particular subgroups. Indeed, tailoring the research process to specific subgroups can help promote the development of more nuanced and relevant research for the participants and community, and is enhanced by involving members of the subgroup in KT strategies (105,106).

Conflicting messaging was identified as a challenge in health-related KT in the Circumpolar North and is also a common challenge in public health promotion globally (107,108). Contradictory messaging can cause confusion about what behavioural changes or actions are appropriate (45). Health researchers working with Inuit communities must find ways to increase the cohesiveness and consistency of messages within and between studies. Active involvement of community stakeholders as liaisons between research projects can encourage discussion across research groups helping researchers increase their own awareness of previous results that were communicated and may be outdated (107,108). This increased coordination among research groups can improve cohesiveness of messages in the future (107,108). Active communication among all affected parties can help facilitate integrated KT (90), improving the comprehension and cohesion of messages in results dissemination KT between researchers, communities, policy-makers and practitioners (97). Lack of time, resources and personnel in the region may be barriers for community stakeholder involvement as research liaisons (45). This may be a place where boundary organizations (e.g. Nunavut Research Institute, Inuit Research Advisors in each settled land claim region of Canada, Arctic Research Consortium of the United States and Greenland Center for Health Research) may be able to play a role in results dissemination of high impact and controversial issues (45).

This study used a systematic, transparent, repeatable approach to identify, evaluate and synthesize literature. Furthermore, 2 independent reviewers screened the literature for relevance (with a high level of agreement) and 2 aggregator databases were searched. While these approaches increase the rigour and robustness of literature reviews, there were several study limitations that should be noted. The small number of health studies that assess KT in Inuit communities in the Circumpolar North that were identified in this review could be a result of an underreporting of KT assessments, a lack of quantity of KT assessments, and/or our search strategy. This review did not seek to capture assessments of KT from key organization websites or other grey literature publications; information from these sources may add to the discussion surrounding KT in Inuit communities.

## Conclusion

The small but growing number of peer-reviewed health-related KT evaluations suggests there is still much to be learned about KT in the Circumpolar North. To learn from past successes or failures in KT, it is strongly suggested that assessment of the KT process takes place, and researchers disseminate the KT assessment results in a format that is accessible and available to future projects and researchers (e.g. peer-reviewed journal article). It may be necessary to rebuild the KT methodological framework for Inuit communities, such that it is grounded in Inuit oral histories and cultural practices, and based in actively attempting to deconstruct power dynamics in the KT process between researchers and research participants. Although community engagement was considered key to successful KT in the majority of the studies included in the review, discussion and discourse on the tensions, challenges and opportunities for improvement in community engagement are necessary. Similarly, assessments of KT should include a critical discussion on the role of community engagement and its effect on successful KT. It is important to develop guidelines for the decision-making process around when, how much and which community member involvement should occur; these guidelines would provide useful direction around community engagement within KT methodological frameworks. Overall, this scoping review was intended to inform future KT methods within Inuit communities and improve future critical reflection and assessments of the KT process.
